# Defibrillator-Induced Tricuspid Abscess Presenting as Diabetic Ketoacidosis and Wound Ulceration

**DOI:** 10.14740/jocmr2404w

**Published:** 2015-12-03

**Authors:** Rafay Khan, Sabrina Arshed, Amar Ahmed, Shuvendu Sen, Abdalla Yousif

**Affiliations:** a Department of Internal Medicine, Raritan Bay Medical Center, 530 New Brunswick Ave., Perth Amboy, NJ 07733, USA

**Keywords:** Endocarditis, Foreign body, Abscess

## Abstract

Right-sided endocarditis is predominantly seen in patients with a history of intravenous drug abuse. However, it is well shown in the literature to be associated with patients containing foreign bodies such as pacemakers, central venous lines, and in those with congenital heart disease. In patients with pacemaker leads and in those with automatic implantable cardioverter defibrillators (AICDs), it is important to suspect foreign body infection when there are signs and indications of bacteremia. When these leads become infected, they can spread the infection to the tricuspid valve resulting in vegetations. The proper management is removal of the infected lead and foreign body along with a prolonged course of antibiotics. However, it is unusual and a relatively rare entity to see foreign body infection resulting from a wound ulcer resulting in not only endocarditis but also abscess formation on the tricuspid valve. Here we report a case of a 60-year-old male with recent AICD placement presenting as diabetic ketoacidosis due to tricuspid abscess formation as a result of a foot ulcer.

## Introduction

Endocarditis of the tricuspid valve due to foreign bodies has become more prevalent and well documented over the recent years. It is abnormal however to see abscess formation as a complication of vegetation formation. Whether the bacteremia resulted from foot ulcers or directly from the automatic implantable cardioverter defibrillator (AICD) remains questionable. When leads become infected, the treatment of choice is sterilization of the tricuspid valve and removal of the foreign body. This case report demonstrates the complications of AICD placement and illustrates an atypical form of endocarditis complicated by abscess formation.

## Case Report

A 60-year-old male with past medical history of hypertension, diabetes, deep venous thrombosis, hyperlipidemia, congestive heart failure with recent AICD and coronary artery disease with history of stent to the right coronary artery presented to the emergency room. Patient was brought in by family in a confused state and history was obtained from his relatives. Patient has been taking insulin for his diabetes; however, his sugar has been high for the last few days. In the emergency room, he was found to have a blood sugar level of 444. Patient was still able to follow and respond to commands appropriately. On physical examination, patient had vital signs of a temperature of 99 °F, blood pressure of 98/68, heart rate of 95, and respiratory rate of 20. Pertinent findings on examination demonstrate mild crackles at lung bases bilaterally. Lower left leg demonstrated an erythematous ulcer with clear discharge. Pulses were weakly palpable in the lower extremities. Laboratory data demonstrated a hemoglobin of 12.6, hematocrit of 38.4, leukocytosis of 15.8, and platelets of 168. Sodium was 128, potassium 4, chloride 91, bicarbonate 21, blood urea nitrogen 94, creatinine 1.5, and glucose 444.

Anion gap was elevated at 17 with serum osmolality of 332 and beta hydroxybutyrate of 3.2. Venous pH was 7.27. Chest radiography was significant for right lower infiltrate. Patient was admitted to the intensive care unit for management of diabetic ketoacidosis likely secondary to sepsis. The sepsis may be attributed to the lower leg ulcer or secondary to pneumonia. Patient was appropriately managed with fluid replacement and insulin drip for his diabetic ketoacidosis. Patient was started on vancomycin 500 mg IV and ceftriaxone 1 g IV for suspected pneumonia. His anion gap improved and he was adjusted to subcutaneous insulin.

**Figure 1:**
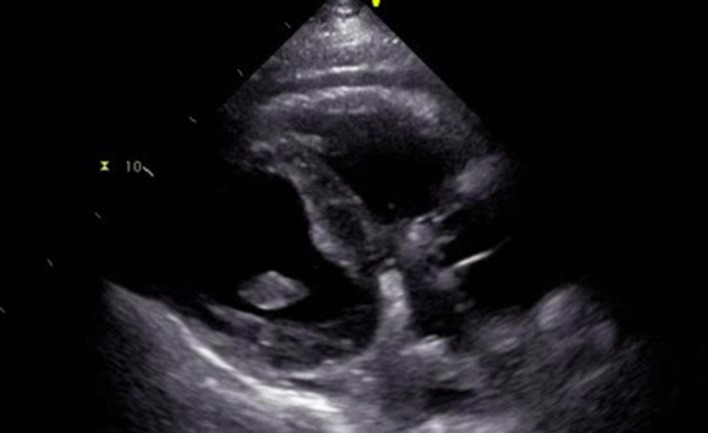
Image findings of TTE without significant demonstration of vegetations.

**Figure 2:**
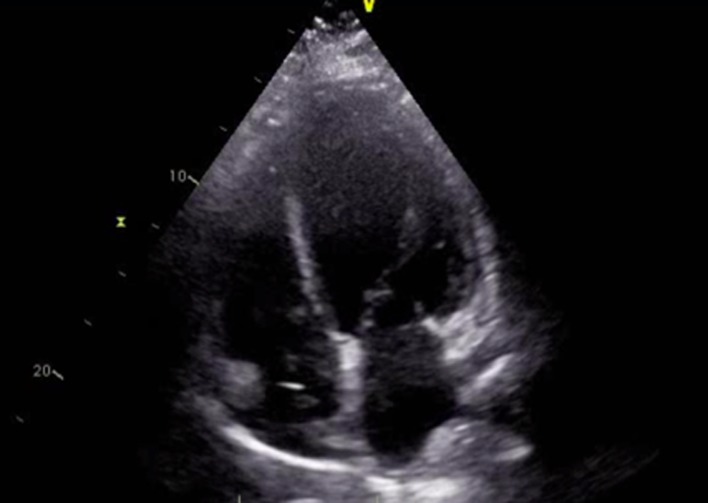
TTE image demonstrating a pattern of global hypokinesis.

Blood cultures returned suggestive of gram positive cocci in clusters which was attributed to his AICD. He initially refused echocardiogram and the source of bacteremia was suspected to be his left foot ulcer. Patient was continued on vancomycin and ceftriaxone at the time. Subculture grew methicillin-resistant *Staphylococcus aureus* which was coagulase positive. After discussion with the patient, he agreed for transthoracic echocardiogram (TTE). TTE was not significant for any vegetations, however, demonstrated an ejection fraction of less than 25% and global hypokinesis with impaired right ventricular systolic function (Fig. 1, 2). However, it did show a mobile echo-density associated with the pacer wire. This study was further evaluated by conducting a transesophageal echocardiogram (TEE). TEE demonstrated significant tricuspid valve disorder suggesting abscess formation rather than thrombus collection. There was a large 1.8 × 2.0 cm highly mobile echogenic structure attached to the atrial side of the anterior leaflet of the tricuspid valve prolapsing to the right ventricle during each diastole. There was a lucent area inside the mass with no flow through. The patient was transferred back to the facility and his AICD was placed for removal due to complications of infection.

## Discussion

There are a significant amount of reports well documenting tricuspid valve vegetation as a result of recent foreign body placement. However, in the literature, there is not a significant description of abscess formation secondary to recent foreign body placement. Whether in this patient, it is a result of recent wound infection which resulted in bacteremia causing abscess formation and endocarditis remains a question and should remain a concern in any physician considering pacemaker or AICD placement in a patient.

The increasing number of patients requiring devices such as AICD and pacemaker has recently increased the number of patients who are at risk for right-sided endocarditis. However, the benefits of such antiarrhythmic devices normally are far more than the complications that it can be associated with. Bacterial endocarditis can result in large vegetations and is invasive to the valves thus destroying the underlying structures and tissues and increasing the risk of emboli, such as pulmonary emboli. Due to these risks, surgical options are available for patients with tricuspid endocarditis. Valve excision results in massive tricuspid regurgitation however, which may limit the quality of life of a patient and even increase the risk of recurrent endocarditis [1].

*S. aureus* causes 50-75% of cases of endocarditis; however, this is most commonly associated with intravenous drug use [2]. Fever, multiple pulmonary emboli, sustained bacteremia, history of intravenous drug abuse, or foreign body presence with the presence of *S. aureus* are signs of right-sided endocarditis [3]. One of the frequently found complications, which can be seen in up to 80% of patients and should prompt TEE investigation, is signs of pulmonary events such as minor atelectasis to large infiltrates, pleural exudates, pulmonary emboli, and cavitations [2].

As stated, one of the common complications associated with ride-sided vegetations is pulmonary emboli. The high risk of vegetations on the tricuspid valve such as these septic pulmonary emboli themselves can result in the formation of respiratory conditions such as pneumonia and itself pulmonary abscess [4, 5]. Uncomplicated tricuspid valve endocarditis has been shown to be successfully medically treated in 80% of cases while in the remaining 20% with large vegetations or poor response to antibiotic penetration, surgical treatment is required [6, 7]. Medical treatment should involve antibiotic treatment covering organisms such as *S. aureus*, streptococci, and enterococci and should also involve penicillinase-resistant penicillins or vancomycin, depending on the local presence of methicillin-resistant *S. aureus* [4, 7]. Although our patient was being managed with vancomycin, the presence of abscess and even the concomitant presentation of recent AICD placement necessitated the requirement for surgical intervention including foreign body removal. With the removal of the AICD or pacemaker, it may be necessary for the patient to undergo valve repair along with the radical removal of the infection and in some cases may require valve replacement. After review of the literature, the early detection, association, and management of abscess formation as a result of foreign body placement have not been well addressed. Whether the presence of a local wound infection should prompt early intervention or delay in AICD or pacemaker placement should be considered. Any sign or source of infection that may result in foreign body associated endocarditis should be managed effectively in order to prevent serious consequences such as abscess formation.

### Conclusion

Abscess formation complicating endocarditis in patients with foreign body placement, especially in those with other sources of infections such as wounds, should remain a concern in any physician considering pacemaker or AICD placement in a patient. Although TTE may show benign findings, it is important to consider TEE in patients with significant bacteremia as even abscess formation may not be detected. The management of abscess formation with medical management alone has been shown to be insufficient and requires both foreign body removal and surgical intervention. However, the treatment modalities involving abscess management still require further research.
